# Phages infecting *Bacteroides thetaiotaomicron*

**DOI:** 10.1128/mra.00760-25

**Published:** 2025-10-30

**Authors:** Joanna P. Steczynska, Kelly P. Williams

**Affiliations:** 1 Systems Biology Department, Sandia National Laboratories 111651, Livermore, California, USA; Queens College Department of Biology, Queens, New York, USA

**Keywords:** phage, gut commensal, bacteroides, bacteroides phage, *Bacteroides thetaiotaomicron*, anaerobes

## Abstract

*Bacteroides thetaiotaomicron* is a prevalent human gut commensal that plays an important role in polysaccharide breakdown and is of interest as a probiotic candidate. We report isolation of lytic phages able to infect *B. thetaiotaomicron*, with nine unique genome sequences belonging to four congeneric species.

## ANNOUNCEMENT

*Bacteroides thetaiotaomicron* (Bt) is a common human gut commensal, with potential therapeutic uses (1). Here, we report isolation of nine phages for this organism that fall into four congeneric species.

Anaerobic digester sewage samples were harvested from the Livermore Water Reclamation Plant (Livermore, CA, USA) wastewater facility on 20 September 2024. Samples were centrifuged, and the supernatants were filtered (0.45 µm) and enriched by adding an equal volume of fresh BHI medium with 1:100 dilution of *B. thetaiotaomicron* VPI-5482. All growth was anaerobic (Don Whitley, A35 chamber, 5% H2), at 37°C. Enriched supernatant was filtered, assayed for plaques on BHI media (37 g/L Millipore), supplemented with yeast extract (5 g/L, Fisher Scientific) and 5% each of newborn calf serum and sheep serum (RmBio). Plaques were clear; 41 were plaque-purified 3 times. Genomic DNA was extracted from high-titer (>10^8^ pfu/mL) stocks using the Norgen Biotek Phage DNA isolation kit, and libraries were prepared using the Illumina DNA prep kit and sequenced using the MiSeq V3 150-cycle kit in paired-end mode using default protocols.

**Fig 1:**
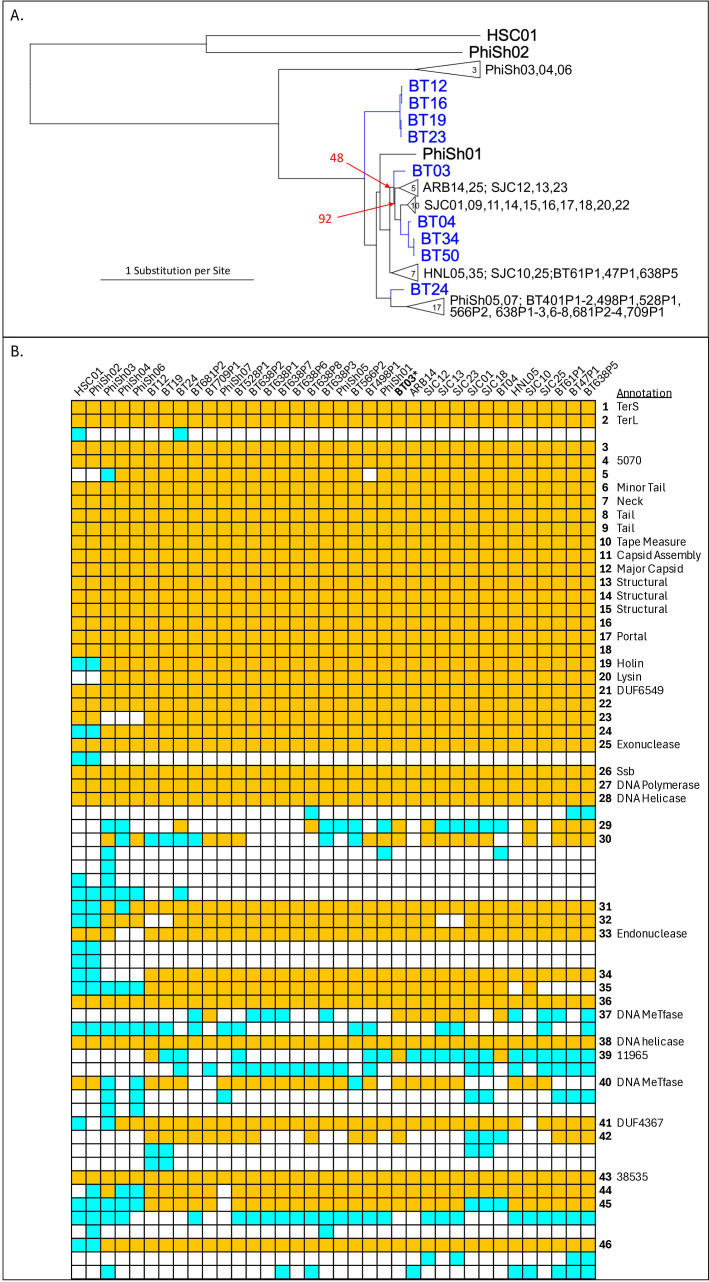
*Bacteroides thetaiotaomicron* phage comparisons. (**A**) Phylogenetic analysis of alpha-cluster *Bacteroides* phages. A 52,418-column alignment was prepared for all genomes (new ones in blue) using MAFFT v7.526 and used to generate an unrooted tree (RAxML-NG with flags --tree pars{25},rand{25} --model GTR + G, with 1,000 bootstraps). All branches had 100% support except for the two marked by arrows. (**B**) Alpha-cluster proteins were clustered as connected components in all vs all BLASTP with a cutoff of 60 bits. Unique orders of the clusters were aligned; those represented in BT03 are colored orange and numbered by the BT03 gene number, others are colored cyan. Protein labels are derived from Pharokka annotations (numerical labels are PHROG IDs that did not assign function; MeTfase: methyltransferase) or from HMMER/HHpred; genes 13–15 are paralogs (separate phylogenies within the same BLASTP cluster).

Reads for 36 phages were assembled using SPAdes (2) v3.15.5. Each assembly yielded a single (~38 kbp) high-coverage contig, except for BT24, whose seven high-coverage contigs were readily assembled based on 55 bp overlaps confirmed with ReadStepper (3). Genomic DNA libraries were re-sequenced using the kit above, in single-read mode to resolve the repetitive gene 21/22 intergentic space in combination with ReadStepper (3). The longer (150 bp) reads also enabled resolution of a problematic tandem repeat region in gene 16, except for BT03 where a tandem 9 bp repeat was truncated at its minimum supported length of 16 copies. Ultimately, all genomes formed closed circles, and the set was reduced to nine unique genome sequences (Table 1). These had close sequence relationships to the 27 genomes of the alpha cluster of previously reported Bt phages (4), to which we added 18 related genomes (names formulated as BTxPy) found at NCBI. Phylogenetic analysis of this alpha-cluster genome set (Fig. 1A) revealed four groups of new phages, represented by BT03, BT04, BT12, and BT24, each considered a separate species within the same genus using VIRIDIC (5), all determined to be tailed phages. There were no matches to any of the 8,630 reference ICTV phages, so taxonomic names were not assigned. Pharokka (6) v1.7.5 (flags: -g prodigal—dnapler) was used for genome annotation, leaving many genes without assigned function. Annotations were verified and further manually supplemented by BLASTP and HMM searches (HHPred https://toolkit.tuebingen.mpg.de/tools/hhpred and HMMER http://hmmer.org/). Default parameters were used for all software unless specified. Gene order comparison (Fig. 1B) revealed a conserved virion gene cluster of structural genes (BT03 g6-18) and a less conserved cluster of replication genes (BT03 g25-46, itself with a conserved core g26-28), as well as lytic and terminase functions. The main variation among new phages was in the presence and type of DNA methyltransferase genes, which likely influence host range. Sequencing of these phages provides an avenue for genetic manipulation of *B. thetaiotaomicron* and better understanding of its viruses.

**TABLE 1:** Characteristics of new unique phage genome sequences

Phage	Size (bp)	Coverage	GC content (%)	Protein genes	tRNA genes	Species cluster	Genbank accession	BioSample accession
BT12	38,363	453.6	45.91	46	0	1	PV788060	SAMN48543848
BT16	38,341	353.6	45.95	46	0	1	PV788052	SAMN48543849
BT19	38,301	299.8	46.22	46	0	1	PV788053	SAMN48543850
BT23	38,304	374.0	46.22	46	0	1	PV788054	SAMN48543851
BT24	38,439	455.3	47.14	48	0	2	PV788055	SAMN48543852
BT03	38,363	497.5	46.03	46	0	3	PV788058	SAMN48543853
BT04	37,773	386.4	46.09	45	0	4	PV788059	SAMN48543855
BT34	37,742	212.4	46.23	45	0	4	PV788056	SAMN48543854
BT50	37,733	223.1	46.20	45	0	4	PV788057	SAMN48543856

## Data Availability

The raw sequence reads were deposited to the NCBI SRA database under BioProject PRJNA1263905. BioSample and GenBank accessions are listed in Table 1 for each phage.
